# Variations in Genomic Testing in Non-small Cell Lung Carcinoma: A Healthcare Professional Survey of Current Practices in the UK

**DOI:** 10.1093/oncolo/oyad134

**Published:** 2023-06-13

**Authors:** Shobhit Baijal, Philip Crosbie, Jackie Fenemore, Ketul Desai

**Affiliations:** University Hospital Birmingham NHS Trust, Birmingham, UK; Manchester University NHS Foundation Trust, Manchester, UK; Division of Infection, Immunity and Respiratory Medicine, Faculty of Biology, Medicine and Health, University of Manchester, UK; The Christie NHS Foundation Trust, Manchester, UK; Novartis Pharmaceuticals UK Limited

**Keywords:** non-small cell lung carcinoma, real world practice, management, treatment, oncogenic genetic testing

## Abstract

This survey aims to understand the current UK practice for non-small cell lung carcinoma (NSCLC) and identify barriers that may impact patient treatment and outcomes. In March–June 2021, 57 interviews were conducted with healthcare professionals involved in the secondary care management of patients with NSCLC. Most respondents performed genetic testing at onsite and non-genomic laboratory hub (GLH) offsite locations. The most common genetic tests were *EGFR* T790M variant (100%), *EGFR* exon 18-21 covered (95%) and *BRAF* (93%). No targeted therapy (TT) available (69%), lack of access to a TT (54%) or excessive molecular testing turnaround times (39%) were the most common reasons for using an immuno-oncology therapy over a TT in the first-line setting. The survey highlights variation in mutation testing practices across the UK, which may impact treatment decisions and contribute to health outcome inequality.

## Teaser

This brief communication reports current practice for non-small cell lung carcinoma in the UK and identifies barriers that could affect patient treatment and outcomes.

## Introduction

The treatment landscape of non-small cell lung carcinoma (NSCLC) has evolved with the discovery of new oncogenic mutations and the introduction of immunotherapy and targeted therapy (TT).^1,2^ In the UK, molecular profiling, including genetic testing, has become standard practice in the management of NSCLC to help determine suitable treatment options. The national genomic testing service is delivered through a network of Genomic Laboratory Hubs (GLHs) to address variations in quality and access to genetic testing across England.^3^ However, the extent to which GLHs are utilized for NSCLC within the UK at the time of the survey is unclear. Despite the standardization of testing infrastructure offered by GLHs, genetic testing rates remain suboptimal across the UK.^4,5^ This is likely due to prolonged turnaround time (TAT), primarily affected by delays in sample request and delivery, analysis and reporting of testing results.^5,6^ The aim of the Lung Adjuvant and Metastatic Pathway Survey (LAMPS) was to understand the current genetic testing practices for NSCLC in the UK.

## Methods

The LAMPS survey was conducted with healthcare professionals (HCPs) known to be involved in the secondary care management of patients with NSCLC between April 2021 and July 2021. HCPs were identified from general and specialist NHS Trusts of varying sizes and levels of research expertise, based at geographically dispersed centers across the UK. The Steering Committee invited 150 potential respondents from an existing UK database of secondary lung cancer care across the UK to participate by email; those who provided consent and were available for interview were contacted by the Novartis Medical Team to take part in the survey. A steering committee consisting of 3 external clinical leads (medical oncologist, respiratory physician, and senior clinical nurse specialist) collaborated with Novartis to develop the structured questionnaire used in the survey. Interviews were carried out remotely by members of the Novartis Medical Team. Descriptive analysis was performed.

## Results

### Demographics and Respondent and Center Profile

In total, 57 HCPs completed the survey, including medical oncologists (40%, n = 23), clinical oncologists (26%, n = 15), clinical nurse specialist (16%, n = 9), pathologists (9%, n = 5), respiratory physicians (5%, n = 3), oncology middle grade (2%, n = 1) and research nurse (2%, n = 1) (Supplementary Fig. S1). Respondents were from geographically distributed centers across the UK (England [84%, n = 48], Northern Ireland [7%, n = 4], Scotland [5%, n = 3], Wales [4%, n = 2]; Supplementary Table S1) with representation from university teaching hospitals (84%, n = 48) and district general hospitals (16%, n = 9).

### Genetic Testing Practices

**Figure 1.**
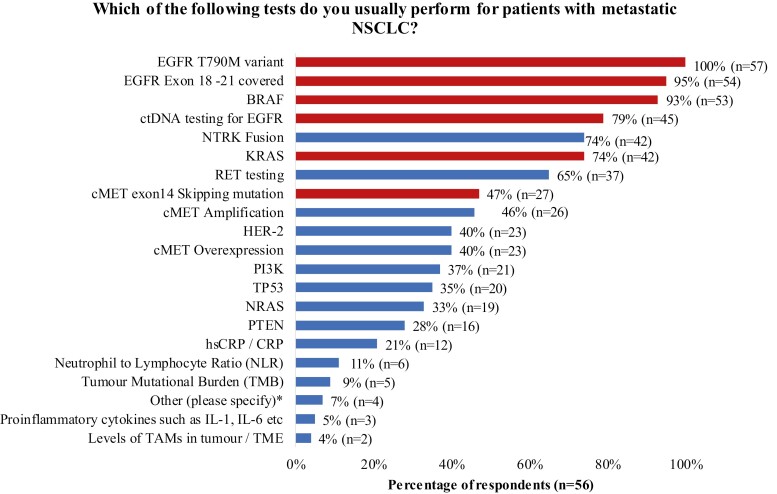
Genetic tests currently performed for patients with metastatic NSCLC at onsite and offsite non-GLH centers. Figure depicts tests performed in real world practice either onsite or at offsite non-GLH locations at the time of survey completion. Red bars in the figure indicate the percentage of respondents currently testing for mutation drivers that feature as part of the standard GLH panel (see Supplementary Tables S2 and S3 for the GLH panel). *Four HCPs responded that other tests were performed, including ALK (*n* = 2), PDL1 (*n* = 2), ROS1 (*n* = 2), and liquid biopsy (*n* = 1). BRAF, B-Raf proto-oncogene; cMET, tyrosine-protein kinase Met; ctDNA, circulating tumor deoxyribonucleic acid; EGFR, epidermal growth factor receptor; HER-2, human epidermal growth factor receptor-2; IL, interleukin; KRAS, Kirsten rat sarcoma viral oncogene homolog; NRAS, neuroblastoma RAS viral oncogene homolog; NTRK, neurotrophic tropomyosin or tyrosine receptor kinase; PI3K, phosphoinositide 3-kinases; PTEN, phosphatase and tensin homolog; RB1, RB transcriptional corepressor 1; RET, REarranged during Transfection proto-oncogene; TAM, tumor-associated macrophages; TP53, tumor protein p53.

All respondents (100%, n = 57) stated that their standard genetic testing panel included *ALK* (anaplastic lymphoma kinase), *PD-L1* (programmed death-ligand 1) and *EGFR* (epidermal growth factor receptor) tests; 96% (n = 55) of respondents included *ROS-1* (ROS proto-oncogene 1) test. Most respondents performed their standard panel genetic testing at onsite (47%, n = 27) and non-GLH offsite (30%, n = 17) locations; 23% (n = 13) of respondents performed genetic testing at a GLH. In the standard testing panel used by onsite and non-GLH offsite centers, the top 3 most common tests were *EGFR* T790M variant (100%, n = 57), *EGFR* exon 18-21 covered (95%, n = 54), and *BRAF* (93%, n = 53) (Fig. 1). However, *cMET* exon 14 skipping (*cMETex14*) mutation was tested by 47% (n = 27) of onsite and offsite non-GLH locations at the time of the survey (Fig. 1) in contrast to GLHs where it forms part of their standard testing panel (Supplementary Table S2; S3).

### Current Treatment Practices

**Figure 2.**
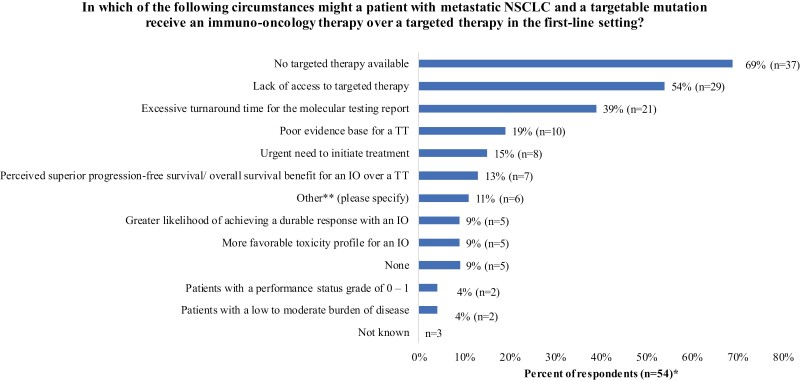
Circumstances in which HCPs might treat a patient with metastatic NSCLC who has a targetable mutation with an immuno-oncology therapy over a targeted therapy in the first-line setting. *Responses are not mutually exclusive. **Other circumstances were reported, including lack of tissue for biomarker testing (*n* = 1); administration problems (n = 1); counter indication to TT (*n* = 1); 2 weeks for genetic test turnaround (*n* = 1); for ROS-1 that is tested sequentially after common mutation (*n* = 1); and EGFR ex20 mutation would receive IO (*n* = 1). EGFR, epidermal growth factor receptor; HCPs, healthcare professions; IO, immuno-oncology; ROS-1, ROS proto-oncogene 1; TT, targeted therapy.

In the survey, HCPs were asked about their preferences toward an effective TT (if available) versus an ­immuno-oncology (IO) treatment in the first-line setting. For patients with high PD-L1 expression and a driver mutation, all respondents (100%, n = 57) preferred to use TT if available over an IO-based treatment in the first-line setting. Additionally, HCPs were asked about their view on current barriers in clinical practice that could impact a treatment choice for a specific patient group. No TT available (69%, n = 37/54), lack of access to a TT (54%, n = 29/54) and excessive molecular testing turnaround times (TAT) (39%, n = 21/54) were the most common circumstances where HCPs might treat a patient with metastatic NSCLC and a targetable mutation with an IO-based therapy over a TT in the first-line setting (Fig. 2).

## Discussion

This survey provides an overview of the genetic testing ­practices for NSCLC across the UK and highlights potential barriers that may impact patient treatment and outcomes. The results showed variation in mutation testing practices; this may impact diagnosis and contribute to health outcome inequality. With the emergence of TT options in recent years,^7,8^ oncogenic driver testing will become increasingly important in selecting appropriate treatments to achieve optimal clinical outcomes. However, less than half of respondents’ centers performed genetic testing for newer targets (*HER2*, *cMET*). Centers (conducting onsite and non-GLH offsite testing) are encouraged to seek access to a broad panel of genetic testing for NSCLC. Moreover, *cMETex14* mutation testing has recently been included in the National Genomic Test Directory and has been introduced in other country guidelines (Supplementary Table S2; S3); it may be adopted across non-GLH centers.

The survey underlines potential barriers to accessing TT (availability, access) as a first-line treatment in the metastatic NSCLC setting, implying unmet medical needs for the discovery of novel molecular targets. Excessive testing TAT was also perceived as a key barrier to accessing TT as a first-line treatment, signifying the urgent need for improvement in availability and efficiency of molecular testing services to inform treatment choices.

These results are consistent with research suggesting variations in molecular testing practices in other European countries.^9,10^ Using external laboratories for biomarker testing is mainly due to a lack of structured access to testing and limited reimbursement/funding, as well a lack of public health system support.^9,10^ However, molecular testing is expected to become more centralized in the future to improve patient access, testing efficiency and quality while reducing costs.^6^

Taken together, our findings highlight the need to address barriers to accessing molecular testing in order to facilitate patient access to molecular-driven therapy and improve clinical outcomes.

Limitations to this survey design include HCP selection methodology, limited geographical representation from the devolved nations, and most participating centers being larger specialist and clinical research focused centers. Responses were based on the interviewed HCPs (limited to a small sample size, n = 57) and may be subject to recall bias. The views of a sample of HCPs involved in the management of NSCLC may not represent the wider UK treating population. There have been changes to real-world practice since the time of the survey (eg, novel TT approved for systemic management^7,8^; *METex14* mutation included in the testing panel [Supplementary Table S2; S3]). This survey was conducted during the first wave of COVID-19 which may influence some of the responses (eg, testing TATs). The descriptive analysis limits data interpretation. Future work may focus on how these survey findings vary by geographic location or respondent speciality.

## Supplementary Material

oyad134_suppl_Supplementary_Figure_S1

oyad134_suppl_Supplementary_Table_S1

oyad134_suppl_Supplementary_Table_S2

oyad134_suppl_Supplementary_Table_S3

oyad134_suppl_Supplementary_Material

## Data Availability

The data underlying this article will be shared on reasonable request to the corresponding author.
