# Supplementary material for: Variations in Genomic Testing in Non-small Cell Lung Carcinoma: A Healthcare Professional Survey of Current Practices in the UK
Source: Oncologist. 2023 Jun 13;28(8):e699–702. doi: 10.1093/oncolo/oyad134 (PMC10400127; doi:10.1093/oncolo/oyad134)
Supplement: oyad134_suppl_Supplementary_Figure_S1 [file oyad134_suppl_supplementary_figure_s1.docx]

**Supplementary Figures**

**Figure S1.** Respondent profile

*Respiratory physicians refer to pulmonologists or specialists in respiratory medicine, and their work involves several fields including but not limited to oncology, infectious disease, and immunology.
