# Supplementary material for: Variations in Genomic Testing in Non-small Cell Lung Carcinoma: A Healthcare Professional Survey of Current Practices in the UK
Source: Oncologist. 2023 Jun 13;28(8):e699–702. doi: 10.1093/oncolo/oyad134 (PMC10400127; doi:10.1093/oncolo/oyad134)
Supplement: oyad134_suppl_Supplementary_Table_S1 [file oyad134_suppl_supplementary_table_s1.docx]

**Supplementary Tables**

**Table S1.** Distribution of participating centers by region

| **Country/Region** | **Centers (n, % n=57)** |
| --- | --- |
| England | 48 (84%) |
| Northern Ireland | 4 (7%) |
| Scotland | 3 (5%) |
| Wales | 2 (4%) |
