# Supplementary material for: Variations in Genomic Testing in Non-small Cell Lung Carcinoma: A Healthcare Professional Survey of Current Practices in the UK
Source: Oncologist. 2023 Jun 13;28(8):e699–702. doi: 10.1093/oncolo/oyad134 (PMC10400127; doi:10.1093/oncolo/oyad134)
Supplement: oyad134_suppl_Supplementary_Table_S2 [file oyad134_suppl_supplementary_table_s2.docx]

**Supplementary Tables**

**Table S2.** Molecular testing guidelines for NSCLC in the UK, EU and US

| **Target gene** | **UK National Genomic Test Directory^a^** | **EU ESMO^b^** | **US ASCO^c^** |
| --- | --- | --- | --- |
| ALK | ✓ | ✓ | ✓ |
| BRAF | ✓ | ✓ | ✓ |
| EGFR | ✓ | ✓ | ✓ |
| ROS1 | ✓ | ✓ | ✓ |
| KRAS | ✓ | ✓^d^ | ✓^d^ |
| MET | ✓ | ✓^d^ | ✓^d^ |
| NTRK | ✓ | ✓ | - |
| RET | ✓ | ✓^d^ | ✓^d^ |
| RB1 | - | - | - |
| ERBB/HER2 | - | ✓^d^ | ✓^d^ |

^a^Sources: NHS England (2022) National Genomic Test Directory for cancer. Available at: <https://www.england.nhs.uk/publication/national-genomic-test-directories/> (Accessed: May 12, 2022).

^b^European Society for Medical Oncology (2023) Metastatic non-small cell lung cancer: ESMO Clinical Practice Guidelines for diagnosis, treatment and follow-up. Available at: <https://www.esmo.org/content/download/347819/6934778/1/ESMO-CPG-mNSCLC-15SEPT2020.pdf> (Accessed: March 16, 2023).

^c^Lindeman, N.I. et al. (2018) ‘Updated molecular testing guideline for the selection of lung cancer patients for treatment with targeted tyrosine kinase inhibitors: Guideline from the College of American Pathologists, the International Association for the Study of Lung Cancer, and the Association for Molecular Pathology’, Arch Pathol Lab Med. 142(3):321-346. doi:10.5858/arpa.2017-0388-CP.

^d^Single gene testing or expanded panel testing.

ALK, anaplastic lymphoma kinase; ASCO, American Society of Clinical Oncology; BRAF, B-Raf proto-oncogene; EGFR, epidermal growth factor receptor; ESMO, European Society for Medical Oncology; EU, European Union; HER, [human epidermal growth factor](https://www.sciencedirect.com/topics/medicine-and-dentistry/gamma-urogastrone) receptor; KRAS, Kirsten rat [sarcoma](https://www.sciencedirect.com/topics/medicine-and-dentistry/sarcoma) [viral oncogene](https://www.sciencedirect.com/topics/medicine-and-dentistry/virus-oncogene) homolog; MET, [hepatocyte growth factor receptor](https://www.sciencedirect.com/topics/medicine-and-dentistry/scatter-factor-receptor); NSCLC, non-small cell lung carcinoma; NTRK, neurotrophic tyrosine receptor kinase; RET, REarranged during Transfection proto-oncogene; ROS1, ROS proto-oncogene 1, UK, United Kingdom; US, United States
