# Supplementary material for: Variations in Genomic Testing in Non-small Cell Lung Carcinoma: A Healthcare Professional Survey of Current Practices in the UK
Source: Oncologist. 2023 Jun 13;28(8):e699–702. doi: 10.1093/oncolo/oyad134 (PMC10400127; doi:10.1093/oncolo/oyad134)
Supplement: oyad134_suppl_Supplementary_Table_S3 [file oyad134_suppl_supplementary_table_s3.docx]

**Supplementary Tables**

**Table S3.** UK National Directory Test Directory for NSCLC (2021/2022)

| **Test name** | **Target genes** | **Recommendations** |
| --- | --- | --- |
| Multi-target NGS panel | | |
| Small variant detection | EGFR, ALK, BRAF, KRAS p.(G12C), MET exon 14 skipping | - Non-squamous NSCLC: full gene panel is recommended, although there may be scenarios where clinicians wish to test other subtypes of NSCLC. - Squamous cell NSCLC: KRAS p.(G12C), MET exon 14 skipping, RET and NTRK gene fusions should be tested. |
| Structural variant detection | ROS1, RET, EML4-ALK, NTRK1, NTRK2, NTRK3, MET 14 exon skipping |  |
| Copy number variant detection to exon level resolution | MET | - Molecular assessment is recommended to aid diagnosis or management. |
| Simple targeted mutation testing | | |
| Small variant detection | EGFR hotspot tumor | - Non-squamous NSCLC: test is usually recommended, although there may be scenarios where clinicians wish to test other unusual subtypes, patient eligible for tyrosine kinase inhibitor therapy, or in rare cases where this cannot be delivered by panel testing. |
|  | EGFR hotspot ctDNA | - Detection of activating EGFR mutations in ctDNA is recommended when biopsy is unavailable and patient otherwise eligible for tyrosine kinase inhibitor therapy. |
| FISH/RT-PCR/Simple targeted mutation testing | | |
| Structural variant detection | ROS1 rearrangement | - Molecular assessment is recommended to aid diagnosis or management. |
|  | RET rearrangement |  |
|  | EML4-ALK | - Non-squamous NSCLC: test is usually recommended, although there may be scenarios where clinicians wish to test other unusual subtypes, patient eligible for tyrosine kinase inhibitor therapy, or in rare cases where this cannot be delivered by panel testing. |
| Copy number variant detection to genome wide resolution | MET | - Molecular assessment is recommended to aid diagnosis or management. |
| Small variant detection | ALK hotspot cDNA | - Non-squamous NSCLC: test is usually recommended, although there may be scenarios where clinicians wish to test other unusual subtypes or where knowledge of ALK mutations would alter management. |

Table S3 is adapted from NHS England (2022) 2021/2022 National Genomic Test Directory for cancer. Available at: <https://www.england.nhs.uk/publication/national-genomic-test-directories/> (Accessed: May 12, 2022).

ALK, anaplastic lymphoma kinase; BRAF, B-Raf proto-oncogene; EGFR, epidermal growth factor receptor; EML4, echinoderm microtubule-associated protein-like 4; FISH, fluorescence in situ hybridization; KRAS, Kirsten rat sarcoma viral oncogene homolog; MET, hepatocyte growth factor receptor; NGS, next-generation sequencing; NSCLC, non-small cell lung cancer; NTRK, neurotrophic tyrosine receptor kinase; RET, REarranged during Transfection proto-oncogene; ROS1, ROS proto-oncogene 1, RT-PCR, reverse transcription-polymerase chain reaction
