# Supplementary material for: Variations in Genomic Testing in Non-small Cell Lung Carcinoma: A Healthcare Professional Survey of Current Practices in the UK
Source: Oncologist. 2023 Jun 13;28(8):e699–702. doi: 10.1093/oncolo/oyad134 (PMC10400127; doi:10.1093/oncolo/oyad134)
Supplement: oyad134_suppl_Supplementary_Material [file oyad134_suppl_supplementary_material.docx]

**Supplementary Materials: LAMPS survey**

**Lung Adjuvant and Metastatic Pathway Survey (LAMPS)**

**INTRODUCTION**

Thank you for taking part in this survey.

The aim of this survey will be to understand the current UK non-small cell lung cancer (NSCLC) patient pathway and how this pathway may change in the next two years, given the ever-evolving treatment landscape. This survey will focus on the following sections: diagnosis, treatment guidelines, genetic testing, the adjuvant setting and the metastatic setting.

Please note that the answers you give will be kept confidential and will be combined with responses received from other survey participants. Neither you nor your institution will be identified when discussing or publishing the aggregated results from this survey.

**Section 1: Interview details**

| **Participant ID:** |  |  |  |
| --- | --- | --- | --- |

| **Interview date:** | DD | MM | YYYY |
| --- | --- | --- | --- |
|  |  |  |  |

| **Interviewer initials** |  |  |  |
| --- | --- | --- | --- |

**Has the consent form been signed by the interviewee?**

Yes

No

**Section 2: Respondent profile**

1. **Please provide your country location**

England

Scotland

Wales

Northern Ireland

1. **Please describe your main practice setting**

District general hospital

University teaching hospital or tertiary centre

Other (please specify) ____________________________

1. **What is your role in the care of patients with NSCLC?**

| ☐ Medical Oncologist | ☐ Clinical Oncologist | ☐ Oncology middle grade |
| --- | --- | --- |
| ☐ Radiologist | ☐ Pathologist | ☐ Other (please specify) ________________________ |

**Section 3: Lung cancer diagnostic investigations**

1. **Which of the following tumour related diagnostic tests for NSCLC are commonly performed? (tick all that apply)**

☐ Positron emission tomography (PET) scan

☐ Endobronchial ultrasound-guided transbronchial needle aspiration (EBUS-TBNA)

☐ Computerized tomography (CT) guided biopsy

☐ Bronchoscopy

☐ Mediastinoscopy

☐ Other (please specify) _____________

☐ Not known

1. **Which of the following physiological assessments for NSCLC are commonly performed at the time of diagnosis? (tick all that apply)**

☐ Electrocardiogram

☐ Echocardiogram

☐ Shuttle walk test

☐ 6-minute walk test

☐ Stair-climbing test

☐ Cardiopulmonary exercise test

☐ Other (please specify) ______________

☐ Not known

1. **Do you have a dedicated lung oncology Clinical Nurse Specialist at your site?**

☐ Yes

☐ No

1. **Do you have a cardiothoracic surgeon specialising in lung cancer at your site?**

☐ Yes

☐ No

1. **Have you completed or do you plan to complete the National Optimal Lung Cancer Pathway (NOLCP) implementation at your site?**

☐ Yes

☐ No

☐ It is planned

☐ Do not know

1. **Do you know on average how many days it takes from first interaction in secondary/tertiary care to a pathologically confirmed and staged diagnosis of NSCLC at your site?**

☐ Yes

☐ No

1. **If you selected ‘yes’ above, on average how many days does it take from referral to a histologically confirmed and staged diagnosis of NSCLC at your site?**

_________ days

**Section 4: Treatment guidelines**

1. **Which guidelines for the management of patients with NSCLC do you follow at your site? (tick all that apply)**

National Institute for Health and Care Excellence (NICE)

Scottish Intercollegiate Guidelines Network (SIGN)

Northern Ireland Cancer Network (NIcaN)

UK Oncology Nursing Society (UKONS)

European Society for Medical Oncology (ESMO)

National Comprehensive Cancer Network (NCCN)

Local guidelines

Other (please specify) _________________

**Section 5: Genetic testing**

1. **In your standard genetic testing panel, which of the following are included for patients with metastatic NSCLC? (tick all that apply)**

ALK

ROS-1

PD-L1

EGFR

Not known

1. **Where is your standard panel genetic testing most commonly performed?**

Onsite

Offsite

Genomic Laboratory Hub

**HOLDER PAGE:** On the following pages we will be asking which of the following tests you usually perform **onsite or offsite** for patients with metastatic NSCLC**.** Please mark the test as **either** onsite **or** offsite.

| BRAF | ctDNA testing for EGFR | RET testing |
| --- | --- | --- |
| EGFR T790M variant | hsCRP / CRP | PTEN |
| EGFR Exon 18 -21 covered | Tumour Mutational Burden (TMB) | NRAS |
| KRAS | Proinflammatory cytokines such as IL-1, IL-6 etc | TP53 |
| cMET Overexpression | Neutrophil to Lymphocyte Ratio (NLR) | PI3K |
| cMET exon14 Skipping mutation | Levels of TAMs in tumour / TME | HER-2 |
| cMET Amplification | NTRK Fusion | Other (please specify…) |

1. **Which of the following tests do you usually perform onsite for patients with metastatic NSCLC? (tick all that apply)**

BRAF

EGFR T790M variant

EGFR Exon 18 -21 covered

KRAS

cMET Overexpression

cMET exon14 Skipping mutation

cMET Amplification

ctDNA testing for EGFR

hsCRP / CRP

Tumour Mutational Burden (TMB)

Proinflammatory cytokines such as IL-1, IL-6 etc

Neutrophil to Lymphocyte Ratio (NLR)

Levels of TAMs in tumour / TME

NTRK Fusion

RET testing

PTEN

NRAS

TP53

PI3K

HER-2

Other (please specify) _____________

Not known

1. **Which of the following tests do you usually perform offsite for patients with metastatic NSCLC? (tick all that apply)**

BRAF

EGFR T790M variant

EGFR Exon 18 -21 covered

KRAS

cMET Overexpression

cMET exon14 Skipping mutation

cMET Amplification

ctDNA testing for EGFR

hsCRP / CRP

Tumour Mutational Burden (TMB)

Proinflammatory cytokines such as IL-1, IL-6 etc

Neutrophil to Lymphocyte Ratio (NLR)

Levels of TAMs in tumour / TME

NTRK Fusion

RET testing

PTEN

NRAS

TP53

PI3K

HER-2

Other (please specify) _____________

Not known

1. **Which additional tests (that you do NOT currently have access to for patients with NSCLC) do you think would be of clinical use? (tick all that apply)**

Not known

None

BRAF

EGFR T790M variant

EGFR Exon 18 -21 covered

KRAS

cMET Overexpression

cMET exon14 Skipping mutation

cMET Amplification

ctDNA testing for EGFR

Tumour Mutational Burden (TMB)

Proinflammatory cytokines such as IL-1, IL-6 etc

Neutrophil to Lymphocyte Ratio (NLR)

Levels of TAMs in tumour / TME

NTRK Fusion

RET testing

PTEN

NRAS

TP53

PI3K

HER-2

Liquid biopsy tests

Other (please specify) ____________________________

1. **Do you retest for mutations at disease progression to metastatic disease?**

Yes (whenever possible)

No

Occasionally

1. **If you selected ‘yes’ or ‘occasionally’ above, which three aberrations do you most commonly re-test for? (select top three)**

ALK

BRAF

ROS-1

PD-L1

EGFR

EGFR T790M variant

EGFR Exon 18 -21 covered

KRAS

cMET Overexpression

cMET exon14 Skipping mutation

cMET Amplification

NTRK Fusion

RET testing

PTEN

NRAS

TP53

PI3K

HER-2

Other (please specify) _________________________

Not known

**Section 6: Adjuvant setting**

1. **What percentage of AJCC stage 2A and above patients undergoing resection for NSCLC have a complete resection (R0)?** ________%
2. **What is the standard adjuvant chemotherapy / radiotherapy regimen offered to squamous NSCLC patients at your site?**

☐ Cisplatin + Vinorelbine

☐ Cisplatin + Gemcitabine

☐ Carboplatin + Vinorelbine

☐ Carboplatin + Gemcitabine

☐ Other (please specify) ______________

1. **What is the standard adjuvant chemotherapy / radiotherapy regimen offered to non-squamous NSCLC patients at your site?**

☐ Cisplatin + Vinorelbine

☐ Cisplatin + Gemcitabine

☐ Cisplatin + Pemetrexed

☐ Carboplatin + Vinorelbine

☐ Carboplatin + Gemcitabine

☐ Carboplatin + Pemetrexed

☐ Other (please specify) ______________

1. **Which three of the following factors will be most likely to influence your decision to use adjuvant therapy in patients with AJCC stage 2A and above NSCLC? (select top three)**

Pathological stage

Age

Patient choice

Comorbidities

Performance status

Smoking cessation

Other (please specify) _________________________

1. **Approximately, what percentage of R0 NSCLC patients recommended for adjuvant chemotherapy are not fit enough to receive treatment?** __________%
2. **Approximately, what percentage of eligible patients decline adjuvant chemotherapy, despite being fit to receive it?** ________%
3. **What are the three most common reasons for patients declining adjuvant chemotherapy? (select top three)**

☐ Patients have not recovered from surgery

☐ Patients are worried about side effects of the treatment

☐ Limited or modest benefit

☐ Travel requirements for treatment

☐ Other (please specify) ________________

1. **Are you currently recruiting R0 patients into any of the following NSCLC adjuvant trials at your site? (tick all that apply)**

☐ Immuno-oncology trials

☐ Targeted therapy trials

☐ Pro tumour inflammation inhibition trials

☐ Chemotherapy trials

☐ Other (please specify) ________________

1. **Are you conducting any neoadjuvant trials in NSCLC at your site?**

☐ Yes

☐ No

**Section 7: Metastatic setting**

1. **Do you know on average, how many days it takes from radiological diagnosis of metastatic NSCLC to the start of systemic treatment at your site?**

Yes

☐ No

1. **If you selected ‘yes’ above, on average how many days does it take from radiological diagnosis of metastatic NSCLC to the start of systemic treatment at your site?**

____________ days

1. **Approximately, what percentage of patients with metastatic NSCLC at your site who are offered first-line systemic treatment decline to receive it?** _______%
2. **Approximately, what percentage of patients with metastatic NSCLC at your site who are offered second / third line systemic treatment decline to receive it?** _______%
3. **What are the three most common reasons for patients with NSCLC declining treatment in the metastatic setting? (top three)**

☐ Patients do not want further treatment

☐ Patients are worried about side effects of the treatment

☐ The impact of receiving treatment on quality of life (QoL)

☐ Limited or modest benefit

☐ Travel requirements for treatment

☐ Other (please specify) ________________

1. **Which of the following factors will be most likely to influence your choice of systemic therapy for patients with metastatic NSCLC? (select top three)**

Efficacy

Safety / tolerability

Previous treatments received

Subsequent treatment options available

Biomarker or mutational test results

Tumour burden

Performance status

Need for rapid response

Impact of treatment on the patient’s quality of life

Patient age

Presence of CNS disease

Comorbidities

Other (Please specify) _____________

**HOLDER PAGE:** On the following pages we will be asking what your standard first-line treatment is for patients with metastatic squamous or non-squamous NSCLC dependent on PD-L1 expression.

The questions will ask you to consider your first line treatment in the following patients;

| **Question** | **NSCLC** | **PD-L1 expression** |
| --- | --- | --- |
| A | Metastatic non-squamous | < 50% |
| B | Metastatic non-squamous | > 50% |
| C | Metastatic squamous | < 50% |
| D | Metastatic squamous | > 50% |

1. **Most commonly, what is your standard first-line treatment for patients with metastatic non-squamous NSCLC who do not have a targetable mutation (PD-L1 <50%)?**

☐ Platinum, pemetrexed and pembrolizumab

☐ Pembrolizumab monotherapy

☐ Chemotherapy (platinum based +/- pemetrexed)

☐ Atezolizumab plus bevacizumab, carboplatin and paclitaxel

☐ Other (please specify) ____________________

1. **Most commonly, what is your standard first-line treatment for patients with metastatic non-squamous NSCLC who do not have a targetable mutation (PD-L1 >50%)?**

☐ Platinum, pemetrexed and pembrolizumab

☐ Pembrolizumab monotherapy

☐ Chemotherapy (platinum based +/- pemetrexed)

☐ Atezolizumab plus bevacizumab, carboplatin and paclitaxel

☐ Other (please specify) ____________________

1. **Most commonly, what is your standard first-line treatment for patients with metastatic squamous NSCLC who do not have a targetable mutation (PD-L1 <50%)?**

☐ Platinum, paclitaxel and pembrolizumab

☐ Pembrolizumab monotherapy676

☐ Chemotherapy (platinum doublet)

☐ Other (please specify) ____________________

1. **Most commonly, what is your standard first-line treatment for patients with metastatic squamous NSCLC who do not have a targetable mutation (PD-L1 >50%)?**

☐ Platinum, paclitaxel and pembrolizumab

☐ Pembrolizumab monotherapy

☐ Chemotherapy (platinum doublet)

☐ Pembrolizumab with pemetrexed and platinum-based chemotherapy

☐ Other (please specify) ____________________

1. **In your experience, approximately what percentage of patients with metastatic NSCLC will complete two years of pembrolizumab in the first line setting?** _____________%
2. **In your experience, approximately what percentage of metastatic non-squamous NSCLC patients tend to go onto Pemtrexed maintenance therapy?**

________________%

1. **In your experience, how long will the average metastatic non-squamous NSCLC patient stay on Pemtrexed maintenance therapy?** ___________weeks
2. **In your experience, approximately what percentage of metastatic non-squamous NSCLC patients tend to go onto combination pembrolizumab-pemetrexed maintenance therapy?**

________________%

1. **In your experience, how long will the average metastatic non-squamous NSCLC patient stay on combination pembrolizumab-pemetrexed maintenance therapy?** ___________weeks
2. **If treating with pembrolizumab in the metastatic NSCLC setting, what is your preferred dosing schedule?**

☐ Every three weeks

☐ Every six weeks

☐ Other (please specify) ________________

1. **If a patient with metastatic NSCLC has high PD-L1 expression and a driver mutation for which an effective targeted therapy is available, do you generally prefer to use targeted therapy over an immuno-oncology therapy in the first-line setting?**

Yes

No

1. **Approximately****,** **what percentage of patients with metastatic NSCLC who are eligible for targeted therapy receive an immuno-oncology therapy in the first line setting?**_____________%
2. **In which of the following circumstances might a patient with metastatic NSCLC and a targetable mutation receive an immuno-oncology (IO) therapy over a targeted therapy (TT) in the first line setting? (tick all that apply)**

No targeted therapy available

Lack of access to targeted therapy

Greater likelihood of achieving a durable response with an IO

More favorable toxicity profile for an IO

Perceived superior progression-free survival (PFS) / overall survival (OS) benefit for an IO over a TT

Poor evidence base for a TT

Urgent need to initiate treatment

Excessive turnaround time for the molecular testing report

Patients with a performance status grade of 0 – 1

Patients with a low to moderate burden of disease

Other (please specify) _________________

**HOLDER PAGE:** On the following pages we will be asking what your standard **second-line** treatment is for patients with metastatic squamous or non-squamous NSCLC dependent on PD-L1 expression.

The questions will ask you to consider your second line treatment in the following patients;

| **Question** | **NSCLC** | **PD-L1 expression** |
| --- | --- | --- |
| A | Metastatic non-squamous | < 50% |
| B | Metastatic non-squamous | > 50% |
| C | Metastatic squamous | < 50% |
| D | Metastatic squamous | > 50% |

1. **Most commonly, what is your standard second-line treatment for patients with metastatic non-squamous NSCLC who do not have a targetable mutation? (PD-L1 <50%)**

☐ Atezolizumab

☐ Docetaxel monotherapy

☐ Nintedanib with docetaxel

☐ Nivolumab

☐ Pembrolizumab

☐ Other (please specify) ____________________

1. **Most commonly, what is your standard second-line treatment for patients with metastatic non-squamous NSCLC who do not have a targetable mutation? (PD-L1 >50%)**

☐ Docetaxel monotherapy

☐ Nintedanib with docetaxel

☐ Chemotherapy (platinum based +/- pemetrexed)

☐ Other (please specify) ____________________

1. **Most commonly, what is your standard second-line treatment for patients with metastatic squamous NSCLC who do not have a targetable mutation? (PD-L1 <50%)**

☐ Nivolumab

☐ Atezolizumab

☐ Pembrolizumab

☐ Docetaxel monotherapy

☐ Other (please specify) ____________________

1. **Most commonly, what is your standard second-line treatment for patients with metastatic squamous NSCLC who do not have a targetable mutation? (PD-L1 >50%)**

☐ Chemotherapy (platinum doublet)

☐ Docetaxel monotherapy

☐ Other (please specify) ____________________

1. ***Hypothetical situation:* Following completion of pembrolizumab in the advanced setting, would you consider re-challenging if patients progress within the following periods (tick all that apply)**

< 6 months

6 < 12 months

≥ 12 months

1. **Are you currently recruiting first-line metastatic NSCLC patients into any of the following types of clinical trials? (tick all that apply)**

☐ Immunotherapy trials

☐ Targeted therapy trials

☐ Pro Tumour Inflammation inhibition trials

☐ Chemotherapy trials

☐ Antibody-drug conjugates trials

☐ None

☐ Other (please specify) _________________

1. **Are you currently recruiting second / third line metastatic NSCLC patients into any of the following types of clinical trials? (tick all that apply)**

☐ Immunotherapy trials

☐ Targeted therapy trials

☐ Pro Tumour Inflammation inhibition trials

☐ Chemotherapy trials

☐ Antibody-drug conjugates trials

☐ None

☐ Other (please specify)_________________

1. **In consideration of the current pathway for NSCLC, where in your opinion is there currently the most unmet need for patients with metastatic NSCLC? (select top three)**

☐ Treatment for patients with poor performance status (ECOG ≥ 2)

☐ When targeted agents have been exhausted for patients with driver mutations

☐ Better biomarkers for choosing immunotherapy-based treatment

☐ Second or third-line therapy options

☐ Better tolerability

☐ More targetable driver mutations

☐ Other (please specify) ________________________________________

**Section 8: Additional questions**

1. **What additional support would be helpful for you as a healthcare professional (HCP) in regard to managing and/or following up patients on new therapies? (tick all that apply)**

HCP adverse event management app

HCP intranet portal

Home care support

Electronic HCP education

Paper HCP education b

☐ None

Other (please specify) _________________

1. **In your opinion, what additional support would be helpful for patients in regard to managing and/or following up patients on new therapies? (tick all that apply)**

Digital patient education

Patient app

Patient intranet portal

Home care support

Electronic patient education

Paper patient education

QR codes on medication packaging

☐ None

Other (please specify) _________________

1. **Within the metastatic NSCLC treatment paradigm, what do you think will be the most exciting developments in the next 12 – 18 months? (tick all that apply)**

KRAS inhibitors

CMET inhibitors

BRAF inhibitors

RET inhibitors

Antibody-drug conjugates

HER-2 inhibitors

PTII (Pro Tumour Inflammation inhibition)

Combinational therapy (targeted therapy and immuno-oncology therapy)

Radiotherapy

Monitoring of disease with liquid biopsy

Antibody-drug conjugates

Personalised adjuvant and neoadjuvant therapies

Improvement in biomarkers for immunotherapy

TIGIT drugs

Other (please specify) _________________

1. **Have any Adverse Events have been reported during the interview?**

**Yes**  **No**

1. **If yes, please state how many?** ____________________
